# Morphological and Genetic Aspects for Post-Mortem Diagnosis of Hypertrophic Cardiomyopathy: A Systematic Review

**DOI:** 10.3390/ijms25021275

**Published:** 2024-01-20

**Authors:** Vincenzo Cianci, Elena Forzese, Daniela Sapienza, Luigi Cardia, Alessio Cianci, Antonino Germanà, Lorenzo Tornese, Antonio Ieni, Patrizia Gualniera, Alessio Asmundo, Cristina Mondello

**Affiliations:** 1 Department of Biomedical and Dental Sciences and Morphofunctional Imaging, University of Messina, Via Consolare Valeria 1, 98125 Messina, Italy; enzocianci.1997@gmail.com (V.C.); elena.forzese.94@gmail.com (E.F.); daniela.sapienza@unime.it (D.S.); lorenzotornese2013@libero.it (L.T.); patrizia.gualniera@unime.it (P.G.); 2 Department of Human Pathology of Adult and Childhood “Gaetano Barresi”, University of Messina, Via C. Valeria 1, 98125 Messina, Italy; luigicardia1@gmail.com (L.C.); antonio.ieni@unime.it (A.I.); 3 Department of Cardiovascular Medicine, Fondazione Policlinico Universitario A. Gemelli-IRCCS, Largo A. Gemelli 8, 00168 Rome, Italy; alessiocianci.1998@gmail.com; 4 Zebrafish Neuromorphology Laboratory, Department of Veterinary Sciences, University of Messina, Via Palatucci snc, 98168 Messina, Italy; agermana@unime.it

**Keywords:** hypertrophic cardiomyopathy, post-mortem analysis, post-mortem genetic test, cardiomyopathy, genotype–phenotype correlation, sudden cardiac death

## Abstract

Hypertrophic cardiomyopathy (HCM) is one of the most common genetic cardiovascular diseases, and it shows an autosomal dominant pattern of inheritance. HCM can be clinically silent, and sudden unexpected death due to malignant arrhythmias may be the first manifestation. Thus, the HCM diagnosis could be performed at a clinical and judicial autopsy and offer useful findings on morphological features; moreover, it could integrate the knowledge on the genetic aspect of the disease. This review aims to systematically analyze the literature on the main post-mortem investigations and the related findings of HCM to reach a well-characterized and stringent diagnosis; the review was performed using PubMed and Scopus databases. The articles on the post-mortem evaluation of HCM by gross and microscopic evaluation, imaging, and genetic test were selected; a total of 36 studies were included. HCM was described with a wide range of gross findings, and there were cases without morphological alterations. Myocyte hypertrophy, disarray, fibrosis, and small vessel disease were the main histological findings. The post-mortem genetic tests allowed the diagnosis to be reached in cases without morpho-structural abnormalities; clinical and forensic pathologists have a pivotal role in HCM diagnosis; they contribute to a better definition of the disease and also provide data on the genotype–phenotype correlation, which is useful for clinical research.

## 1. Introduction

Sudden cardiac death (SCD) is defined as an unexpected natural death due to a cardiac cause that occurs within a short period of time, generally within the first hour from the onset of symptoms, or in subjects without any symptoms 24 h previously [1,2].

The annual incidence of SCD in Europe and North America is 50–100/100,000 persons [3,4,5]; Asia has an estimated value of 40/100,000 persons [6].

The main cause of sudden cardiac death is coronary artery disease (CAD), with an accepted proportion of all SCDs due to CAD of ≈80%, and SCD accounts for ≈50% of all CAD-related deaths [7]. The other causes of SCD are cardiomyopathies, myocarditis, valvular disease, and channelopathies. Specifically, the distribution of the SCD causes varies with age, resulting in the fact that in young people SCD is mostly due to diseases that are categorizable into those which are structural or those which are purely arrhythmogenic [8]. The structural causes include the inherited cardiomyopathies, among which hypertrophic cardiomyopathy (HCM) is reported to be the most common cause of SCD in young adults, but it is also a major cause of morbidity and mortality in the elderly [8,9,10].

Hypertrophic cardiomyopathy (HCM) is defined as the presence of increased LV wall thickness (with or without RV hypertrophy) or mass that is not solely explained by abnormal loading conditions [11]. The estimated prevalence of HCM is 1:500 in a healthy young population. The penetrance is incomplete and age-related; thus, HCM can present at any age [12,13,14]. Some authors report that HCM is the second most frequent cause of sudden cardiac death, after atherosclerotic coronary artery disease [15].

HCM is an autosomal dominant disease, showing a familial pattern of inheritance in approximately one-half of cases, while in the other cases, sporadic or de novo mutations are found [10]. The main genes involved in the onset of the pathology genes encode for different cardiac sarcomere proteins. These proteins can be associated with a specific macroscopic and microscopic phenotype, including asymmetrical hypertrophy, which is mostly observed in the interventricular septum, the left ventricular cavity reduction, and/or the myocyte disarray with interstitial fibrosis [11,12]. However, HCM shows a relevant heterogeneity in both phenotype and genotype; in fact, different cardiac morphologies and different levels of severity in disease and prognosis can arise from different genes or different mutations in the same gene. This variety is confirmed by cases showing identical genetic mutations in which diverse phenotypic patterns are described between unrelated families or even among affected individuals from the same family [16].

Clinical and judicial autopsy can provide an important contribution in the characterization of the genetic and phenotypic features of HCM. In fact, the post-mortem analysis can be a source of evidence for the genotype–phenotype correlation, especially since the so-called molecular autopsy assumed a strong role in the assessment of cardiovascular diseases with a genetic background that were causes of SCD. This should be stressed even more because some cases reveal normal cardiac macroscopic and microscopic findings. Furthermore, the European Society of Cardiology (ESC) guidelines recommend the performance of post-mortem genetic analysis in suspected SCD cases which can be related to a specific inheritable channelopathy or cardiomyopathy [17].

Therefore, the aim of this paper is to conduct a systematic review of the forensic/post-mortem literature describing HCM cases and to provide an overview of the macroscopic, microscopic, radiological, and molecular data that are useful for a confident post-mortem diagnosis. Focused attention is paid to the genetic aspects and to the analysis of any correlation between the morphological variant and the genotype patterns.

## 2. Materials and Methods

The systematic review was conducted according to the PRISMA guidelines.

The review was conducted by employing the PubMed and Scopus databases. A search was made for articles published up until August 2023 and written in the English language. For a broader search, the key terms used were “hypertrophic cardiomyopathy” or “HCM” in association with the terms “forensic” or “post mortem” and “gross examination” or “histopathology” or “imaging” or “genetics”. The main rule for article selection was the identification in the titles and/or the abstracts of words/concepts indicating the analysis of HCM cases from a forensic/post-mortem point of view. The whole article was then read if the abstract suggested it could potentially meet the inclusion criteria. Articles were excluded by title, abstract, or full text if they did not deal with the topic. Review articles were also excluded, as were editorials, letters to the editor, and articles on HCM related to genetic syndromes. The works considered relevant were analyzed in-depth; the focus was on the gross and histological findings, radiological evidence, and genetic data. Bibliographic references from the selected papers were also screened for possible additional inclusions of the relevant literature that showed that it met the review inclusion criteria.

The process for data collection involved the study selection and data extraction. In particular, to reduce the risk of bias, the systematic analysis was conducted as follows. Three authors independently performed the first step of article selection by title and/or abstract; then, the works with an eligibility agreement from the researchers were read in their entirety. Finally, the articles that obtained consensus from two authors regarding satisfaction of the inclusion criteria were included in the review. Data extraction was performed by two investigators; the data were then verified by two other authors.

## 3. Results

**Figure 1:**
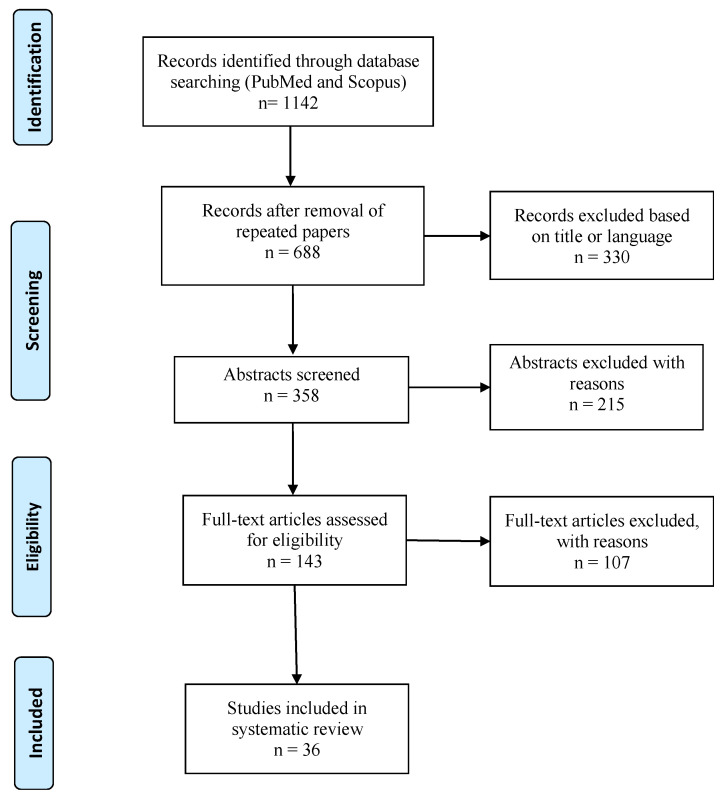
Flowchart of literature selection process.

Figure 1 shows the paper selection results. The selection process led to the inclusion of 36 articles, including case reports, case series, and retrospective observational studies. The general information from the included papers (authors, publication year, type of article, and provided data on HCM) is reported in Table 1 and Table 2.

### 3.1. Risk of Bias

It must be noted that this review included studies that were published within a long time frame; thus, despite our efforts to uniformly evaluate the existing literature, the data obtained from the study should be interpreted by taking into account both the improvement of the knowledge on HCM and the evolution of these cardiomyopathy definitions over the years. Table 1 offers an overview of the main heterogeneities related to the type of study, sample characteristics (i.e., age, sex, and number of cases), and morphological data described (i.e., heart weight and wall thickness). Finally, it must be noted that the genetic data could have an intrinsic risk of bias due to the different gene analysis panels (i.e., number of genes that are screened) used by each research group.

### 3.2. Gross Findings

The macroscopic findings of the literature review are reported in Table 1. Left ventricular hypertrophy is the main evidence; it is categorized as either asymmetrical or symmetrical hypertrophy [18,19,20,21,22,23,24,25,26,27]. The asymmetrical hypertrophy was the most common, and it was frequently described at the ventricular septum, as a disproportionate thickening when compared with the left ventricular free wall [18,20,24,25,27]. In some cases, the septal thickening involved the subaortic basal anterior septum (classical anatomic form) determining the narrowing of the left ventricular outflow tract [18,20,24,25]. This was also associated with a subaortic mitral valve lesion characterized by the valve leaflets thickening due to fibrosis [18,19,21,23]. The hypertrophic subaortic septum can also show fibrous areas with variable size [19,20,21,26]. Some cases of symmetrical HCM were described as concentric thickening of the left ventricle with reduced cavity dimensions [19,20,22,23,24,25].

The right ventricle was described as normal or as having a variable degree of hypertrophy and cavity size decrease [18,19,23].

A macroscopic pattern of ventricular dilatation similar to that of dilated cardiomyopathy (DCM-like), due to HCM progression, was described [28,29,30]. The dilatation was found in the left ventricle or in both ventricles with broad areas of fibrosis. Left ventricle hypertrophic sites can coexist [20,30].

In both the symmetrical and asymmetrical forms, as well as in DCM-like HCM, were also found whitish and scattered scars related to fibrosis replacement in both ventricles [18,23,28,29,30]. Finally, in one study there were cases showing a left anterior descending coronary myocardial bridge [22].

### 3.3. Microscopic Findings

Myocyte hypertrophy and disarray, together with fibrosis, were the main hallmarks described in the histological analysis [18,19,20,21,22,23,24,25,26,27]. The hypertrophic myocytes were described as having abnormal branch or triradiate and stellate forms [19,21] and abnormal nuclei, which were described as enlarged, hyperchromatic, or of atypical shapes [19,21,23,24,25]. The myocyte disarray consisted in altered fiber organization, which was characterized by the foci of adjacent hypertrophied myocytes being oriented perpendicularly or obliquely. In the asymmetric form, the disarray was prominent in the septum, even if it was also found in the left ventricular free walls [18,21,25]. Maron et al. [31] performed a study on 51 cases, in which the cell disorganization was classified into four types (I-A, I-B, II-A and II-B). The analysis revealed the presence of type I-A and/or type I-B septal disorganization in all cases, respectively: type I-A included areas of myocardium that generally had a small size, in which adjacent cardiac muscle cells were aligned perpendicularly or obliquely to each other and usually formed tangled masses or “pinwheel” configurations; type I-B included relatively broad bundles of muscle cells (the cells were normally arranged) oriented at oblique or perpendicular angles to each other. Moreover, the quantification of disarray extension revealed that it was at least 5% of the relevant part of the tissue section (specifically, in 56% of the cases the disorganization was observed in 25% or more). This value was described by other researchers, who reported a disarray greater than 5% in the ventricular septal section and a mean disarray value equal to 52% [32]. However, the research group highlighted that the value of 5% may be too low for the diagnosis of HCM and may lead to a small number of false-positive diagnoses. Basso et al. [22] also quantified the extent of septal disarray, describing a mean percentage area value of 30 ± 16%. A study has also investigated the relationship between myocyte disorganization extension and the left ventricular wall thickness, concluding that the two morphologic aspects did not appear to be directly related [33]. Hypertrophic myocytes and disarray were also observed in the DCM-like form [28,29,30].

Fibrosis was found with variable degrees; frequently, it was more severe in the ventricular septum, pericellular, and perivascular areas [18,19,20,21,22,23,24,25,26,27]. It has been described as massive in several sites of the left ventricle in the DCM-like forms [28,29,30].

In some cases, pathological changes in the intramyocardial arteries were found, such as increased thickness of the wall due to hypertrophy of the media and intimal, causing lumen narrowing [18,22,23,29,30]. This was associated with subacute or acute signs of myocardial ischemia [18,22].

A summary of the microscopic findings described in each of the included articles is reported in Table 1.

**Table 1:** Summary of the main macroscopic and microscopic data.

Authors	Sample (*n*.)	Heart Weight	HCM Type	Thicknesses	Gross Findings	Microscopic Findings
Roberts et al. [18]	37 (mean 44 years)	dnr	Asymmetric (32 cases)	dnr	Clear LV outflow obstruction associated with hypertrophy of LVW with similar degree (17 cases); reduction in both ventricular cavities (36); thickness of mitral valve leaflets (26 cases); dilatation of both atria (36 cases); endocardial scars; foci of myocardial necrosis (1 case)	Interstitial fibrosis of varying degrees in LVW and ventricular septum; varying degrees of disorientation of myocardial fibers in the ventricular septum; increased number in the ventricular septum; increased thickness of the walls of intramural coronary arteries (1 case)
Davies et al. [19]	47 (13.87 years)	180–740 g	Asymmetric (30); symmetric (17 cases)	dnr	Small LV cavity; band-like endocardial fibrous thickening in interventricular septum extending for 2 to 4 cm below the aortic valve with well-defined lower edge having the configuration of a mirror image of the mitral aortic surface; flat fibrous thickening of the aortic surface of the anterior mitral cusp; bullet-like shape of papillary muscle; RV mass varied from normal to grossly increased	Thick and abnormally branched myofibers, disorganized architecture, fibrosis, and whorl formation; endocardial superficial and dense collagenous and elastic fibrotic areas at both subaortic interventricular septum and aortic surface of anterior mitral cusp
Pomerance et al. [20]	15 (>60 years)	mean 506 g	Asymmetric (5 cases); symmetric (10 cases)	dnr	Hypertrophy of the LVW and interventricular septum; band of fibrous endocardial thickening over the upper part of the interventricular septum	Areas of disorganized myofiber architecture with short broad abnormally branched fibers; fibrosis; endocardial ‘pockets’, jet lesions, and endocardial friction lesions
Edwards et al. [21]	1 (13 years)	690 g	Asymmetric	Septum 37 mm; LVW at mitral papillary muscle 26 mm; LVW at base 20 mm; RV 5 mm	Small LV cavity; hypertrophied mitral papillary muscle; endocardial fibrosis at subaortic outflow tract; mitral valve anterior leaflets firm and thickened	Abnormally oriented myocytes in interventricular septum, frequent in the LVW, infrequent in RV free wall; variable size of myofibers with bizarre triradiate and stellate forms, atypical hyperchromatic macronuclei; diffuse interstitial fibrosis in ventricles
Basso et al. [22]	19 (mean 23 years)	mean 457 ± 202 g	Asymmetric (14 cases); symmetric (5 cases)	Septum mean 21.8 ± 6.7 mm	Mirror image septal subaortic plaque with anterior mitral valve leaflet thickening (6 cases); fibrotic scars within the ventricular septum (11 cases); LAD myocardial bridge (4 cases)	Ventricular septum disarray (mean percentage area 30 ± 16%); tiny interstitial myocardial fibrosis; variable degrees of medial hypertrophy–dysplasia and intimal hyperplasia of small intramural coronary arteries; multifocal, patchy signs of acute–subacute myocardial ischemia (coagulative necrosis, neutrophilic infiltrate, myocytolisis, and granulation tissue healing) in septal myocardium (14 cases)
Phadke et al. [23]	14 (all ages)	mean 514 g	Asymmetric (7 cases); symmetric (7 cases)	Septum mean 1.7 cm; free wall 1.2 cm	Variable localization and degree of the asymmetric hypertrophy (dnr); mild to moderate RV hypertrophy (10 cases); mild to moderate chamber dilatation (7 cases); streaky fibrosis (4 cases); anterior mitral leaflet mirror image with leaflet thickening (3 cases)	Striking myofiber hypertrophy with nuclear enlargement and hyperchromasia (14 cases); box and cigar shaped nuclei with perinuclear clearing; myofiber disarray (>5%); obliterative small vessel disease (7 cases); myofibers waviness, smudging and vacuolation; interstitial, interfibrillar and less frequent perivascular fibrosis.
Ahmad et al. [24]	15 (mean 26.6 years)	mean 440 g	Asymmetric (9 cases); symmetric (6 cases)	Septum mean 23.2 mm; LV wall 22.5 mm	Thickening of LV outflow tract (4 cases); obliterative small vessel disease with perivascular fibrosis (5 cases)	Hypertrophy of myofiber with nuclear enlargement and hyperchromasia; nuclei with a cigar or typical box shape; significant myofiber disarray in both septum and free left ventricular wall; patchy interstitial fibrosis.
Kundu et al. [25]	2 (11 and 13 years)	340 g; 450 g	Asymmetric (case 1); symmetric (case 2)	Case 1: LVW 15 mm—septum 18 mm—RV 8 mm; Case 2: Septum 21 mm—RV 6 mm	Case 1: narrowing of LV outflow tract; patchy areas of fibrosis in interventricular septum. Case 2: LV chamber reduced	Hypertrophied myocardium with myofiber disarray and a whirling pattern around areas of fibrosis in LV and interventricular septum; short myofibers, with anisonucleosis and perinuclear vacuolation in some; thickened walls of intramyocardial blood vessels; focal areas with replacement fibrosis in endocardium.
Williams et al. [26]	4 (38, 38, 57, 67 years)	890, 980, 450, 600 g	Asymmetric	Case 1: LVW 24 mm—RV 6 mm—Septum 36 mm; Case 2: LVW 20 mm—RV 8 mm—septum 33 mm; Case 3: LVW 13 mm—RV 3 mm septum 13 mm with anteroseptal region 30 mm; Case 4: LVW 11 mm—RV 2 mm—19 mm	Case 1: no other data; Case 2: heart dilated and asymmetrically hypertrophied; Case 3: no other data; Case 4: interventricular free wall asymmetric thickening, patchy subendocardial fibrosis at subaortic region of the outflow tract	Case 1: perivascular fibrosis; thickening of epicardial and intramyocardial blood vessels; septal myocyte hypertrophy with disarray. Case 2: myocyte hypertrophy, disarray, and interstitial fibrosis. Case 3: myocyte disarray. Case 4: myocyte disarray; perivascular and interstitial fibrosis.
Takei et al. [27]	1 (20 years)	dnr	Asymmetric	dnr	LV hypertrophy with asymmetric hypertrophy of the interventricular septum	Marked hypertrophy of the myocytes, disorderly pattern of myocardial bundles and high degree of interstitial fibrosis in the ventricular septum and the posterior wall of the LV, focal area of myocardial fibrosis
Yamadori et al. [28]	1 (60 years)	450 g	DCM-like	AVS 9 mm; PVS 13 mm; AVW 20 mm; LVW 8 mm; PVW 10 mm	Dilatation of both ventricles; several fibrotic areas in septum and LV and RV walls; myocardial disappearance in AVW; dilatation of both atria	Massive disappearance of myofibers and scar-like fibrosis in posterior interventricular septum and LVW; small patches of fibrosis in both ventricles and papillary muscles; diffuse myocyte hypertrophy; disarray at interventricular septum, posterior wall of both ventricles, LV antero-lateral wall.
Horita et al. [29]	1 (33 years)	610 g	DCM-like	AVW 8 mm; PVW 22 mm; RV 7 mm	Hypertrophy of the posterior and interventricular septal wall; significant decrease in LV apical wall thickness; dilatation of LV cavity; significant fibrosis of LV and RV walls	Massive transmural fibrosis of the AVW; bizarre myocardial hypertrophy with disorganization in PVW and interventricular septum; intramural small artery intimal thickening with severe narrowing
Dettmeyer et al. [30]	1 (8 years)	270 g	DCM-like	LVW (basal region) 9 mm; septum 13 mm	Dilated ventricles with diffuse endocardial fibrosis; endocardial thickening of the ventricular septum below the aortic valve; slight thickness of mitral valve	Extensive fibrous scarring and microfocal myocytolysis in LV subendocardium and papillary muscles; scarring suggestive of ischemia associated with irregular arrangement of fibers and dysplastic vessels with wall increased thickness; extensive and partly bizarre hyperchromatic nuclei; extremely hypertrophic cardiomyocyte groups
Maron et al. [31]	54 (mean 33 years; range 11–70 years)	mean 535 g	dnr	Septum mean 26 mm	dnr	Fifty-one cases with myocytes disorganization in more than 5% of the tissue section: areas of myocardium generally having small size in which adjacent cardiac muscle cells were aligned perpendicularly or obliquely to each other (type I-A); and relatively broad bundles of muscle cells (the cells were normally arranged) oriented at oblique or perpendicular angles to each other (type I-B)
Rose et al. [32]	27 (dnr)	551 ± 117 g	Asymmetric in 69%	Septum 23.9 ± 5.6 mm	dnr	All cases: disarray greater than 5% in ventricular septal section and a mean disarray value equal to 52%
Maron et al. [33]	31 (mean 34 years; range 11–61 years)	adults 592 ± 171 g; children 270 to 530 g	Asymmetrical	Septum mean 22 mm	dnr	Septum: disorganization from 5 to 24% in 26% of the cases, 25 to 49% in 19%, and ≥50% in 42%. AVW (in 25 cases): disorganization from 5 to 24% in 29%, 25 to 49% in 6%, and was particularly extensive, involving ≥50% in 36%. PVW (in 23 cases): disorganization from 5 to 24% in 19%, 25 to 49% in 19%, and ≥50% in 16%

LV: left ventricle; RV: right ventricle; LVW: lateral ventricular wall; AVS: anterior ventricular septum; PVS: posterior ventricular septum; AVW: anterior ventricular wall; PVW: posterior ventricular wall; DCM: dilated cardiomyopathy; LAD: left anterior descending coronary artery.

### 3.4. Imaging

Post-mortem magnetic resonance (PMMR) is reported to be a useful tool because it offers data about cardiac features, supporting the diagnosis of HCM. Firstly, MR showed ventricle hypertrophy and dilation; then, it allowed the calculation of heart weight using either the multiplication of the myocardial volume by the coefficient 1.05 g/cm^3^ or mass analyzing software [34].

Other HCM morphological features, which are demonstrated by PMMR, are the intramyocardial bridge, ventricular crypts, and anomalies of the papillary muscles and sub-mitral apparatus [35]. Some researchers [36] analyzed the use of PMMR to consider the effect of myocardial rigor mortis. They analyzed the standard deviation (SD) of wall thickness (WT) in living patients, simulating rigor mortis (by a mid-diastolic cardiac phase of the CMR cine-image); then, the results were validated in the PMMR cases using the study groups formed, respectively, from HCM, CAD, and non-cardiac cases. The analysis concluded with an SD of WT cut-off of 2.4 to identify HCM with PMMR.

On the other hand, as was described, in a sample of two cases with PMMR data supporting the diagnosis of HCM, there was one false positive (radiologically showing hypertrophy of mid-posterior wall at the level of papillary muscle) because the disease was not confirmed at autopsy [37].

The left ventricular hypertrophy and the asymmetrical septal hypertrophy associated with hypervascularity in the upper portion of the septum were also described using post-mortem computed tomography angiography (PMCTA) [27].

### 3.5. Molecular Autopsy

The genetic test, as a fundamental investigation for the post-mortem assessment of HCM, was reported in several studies [26,38,39,40,41,42,43,44,45,46,47,48,49,50,51,52,53]. The related data obtained from the articles are summarized in Table 2. Genes codifying sarcomeric protein were the most frequently involved, with a prevalence for the mutation of MYBPC3 (Myosin-binding protein C) [26,39,40,41,43,44,45,47,48,49,50,52], in which 12 pathogenic or likely pathogenic variants were reported (c.2221delG; c.3491-2A>T; p.Gln969Ter; p.Phe295SerfsTer5; p.Gly278GlufsTer22; c.649A>G; c.150C>A; c.2003G>A; c.1504C>T; c.442G>A; p.Arg726Cys; c.2441_2443del). Then, mutations were observed at MYH7 (Myosin heavy chain 7) [38,39,40,41,43,44,46,47,48,49,50] with nine variants classified as pathogenic or likely pathogenic (c.1325G>A; R249Q; c.5120T>C; (c.3134G>T; Thr446Pro; Phe468Leu; p.Arg652Lys; c.2105T>A; c.G2348T). Other pathogenic and likely pathogenic variants were found at MYH6 (Myosin heavy chain 6; c.4193G>A) [43], ACTN2 (Actinin α2; c.355G>A) [51], TNNT2 (Troponin T type 2; c.517_519de) [50], TPM1 (Tropomyosin 1α¸ c.656A>T) [53], and TTN (Titin; c.48163C>T) [43].

Two studies analyzed the phenotype–genotype correlation. Hata et al. [47] reported HCM-related rare variants in cases with a disarray >5%: the pathogenic/likely pathogenic variants p.Arg470Gln of MYBPC3 observed in one case and pThr1046Met of MYBPC3 found in two cases showed, respectively, 8.5%, 12.5%, and 15.2% of disarray, while variants of uncertain significance were reported in five cases. However, the authors concluded that it was impossible to genetically determine the clinical appearance and outcomes of HCM considering the large number of factors (i.e., genetic and environmental) that can affect the morphological, histological, and clinical phenotype. On the other hand, Williams et al. [26] described two predicted protein-truncating variants (PTVs) in MYBPC3 (p.Gly278GlufsTer22 and p.Phe295SerfsTer5) associated with high heart-weight-to-BMI ratios and death under age 40, supporting the PTV correlation with a more severe HCM phenotype.

Finally, in some cases, variants that were likely pathogenic or pathogenic were found in the absence of morphological cardiac abnormalities indicating HCM [38,48,50,52,53].

**Table 2:** Synthesis of the main genetic results.

Authors	Sample (*n*)	Gross/Microscopic Data	Gene/Nucleotide or Amino Acid Change	Mutation Type	Phen.
Loporcaro et al. [38]	1 SUDS	-	MYH7 (R249Q)	MM	P
Maeda et al. [39]	11 HCM (6)	+	Case 1: MYBPC3 (IVS3 + 41 G/C; Gln998Glu)	SM/MM	nr
		+	Case 2: MYH7 (Arg1974Gln); MYBPC3 (IVS2 + 55 T/C); MYBPC3 (Ser25Ser)	MM/SM/SiM	nr
		+	Case 3: MYBPC3 (Thr1046Met)	MM	nr
		+	Case 4: MYH7 (Ile989Ile); TNNT2 (IVS15 + 61 A/G); TNNT2 (Thr284Thr)	SiM/SM/SiM	nr
		+	Case 5: MYBPC3 (IVS3 + 41 G/C); MYBPC3 (Ser593fs:1)	SM/FM	nr
		+	Case 6: MYBPC3 (Arg1073Pro); MYBPC3 (IVS3490 G/A)	MM/SM	nr
Allegue et al. [40]	37 SCD of which 23 with autopsy cardiac anomalies (2)	+	Case 1: MYH7 (c.2945T>C)	nr	nr
		+	Case 2: MYBPC3 (c.2440_2442delAAG)	nr	nr
Larsen et al. [41]	12 HCM (1)—8 HCM/DCM (2)	+	Case 1 (HCM): MYBPC3 (c.1564G>A)	nr	B
		+	Case 2 (HCM/DCM): MYH7 (c.1325G>A)	nr	LP
		+	Case 3 (HCM/DCM): MYBPC3 (c.495G>C)	nr	B
Kane et al. [42]	1 HCM	+	TNNI3 (Arg204His)	nr	nr
Hertz et al. [43]	5 HCM (2)—52 with non-diagnostic structural findings (5 with hypertrophy or hypertrophy/fibrosis)	+HCM	Case 1 (HCM): HCN4 (c.1403C>T) *; MYBPC3 (c.649A>G; c.150C>A)	MM/MM/MM	LP
			Case 2 (HCM): TTN (c.48163C>T)	MM	LP
			Other cases: MYH7 (c.5120 T>C); DSP (c.8033G>A); KCNA5 (c.98A>T); MYH6 (c.4193G>A); AKAP9 (c.2146G>C)	MM/MM/MM/MM/MM	LP
Corbett et al. [44]	15 HCM (4)	+	Case 1: MYBPC3 (p.Gly1093Cys)	nr	NV
			Case 2: MYBPC3 (p.Arg668His)	nr	LP
			Case 3: MYBPC3 (p.Arg502Trp)	nr	LP
			Case 4: MYH7 (p.Arg1045Leu)	nr	LP
Williams et al. [26]	4 HCM	+	Case 1: MYBPC3 (p.Gly278GlufsTer22)	FM	P
			Case 2: MYBPC3 (p.Phe295SerfsTer5)	FM	P
			Case 3: MYBPC3 (p.Gln969Ter)	NSM	P
			Case 4: MYBPC3 (c.3491-2A>T)	SM	P
Gaertner-Rommel et al. [45]	1 HCM	+	MYBPC3 (c.442G>A); FHL1 (c.267C>A)	MM/DNM	LP/P
Liu et al. [46]	18 HCM (9)	+	Cases 1 to 8: MYH7 (Thr446Pro)	MM	P
			Case 9: MYH7 (Phe468Leu)	MM	P
Hata et al. [47]	15 HCM (8)	+	Case 1: MYBPC3 (p.T1046M)	MM	LP-P
			Case 2: MYBPC3 (p.R470Q)	MM	LP-P
			Case 3: MYH6 (p.D629N)	MM	VUS
			Case 4: PRKAG2 (p.G75A)	MM	VUS
			Case 5: MYBPC3 (p.T1046M); MYBPC3 (p.R1138C)	MM/MM	LP-P/VUS
			Case 6: CAV3 (p.R148Q)	MM	VUS
			Case 7: MYBPC3 (p.E334K)	MM	VUS
			Case 8: MYH7 (p.941H)	MM	VUS
Ripoll-Vera et al. [48]	5 HCM (4), 30 SUDS (4)	+(HCM)	Case 1 (HCM): MYH7 (p.Arg652Lys)	nr	P
			Case 2 (HCM): LMNA (p.Arg439Cys) ^	nr	LP
			Case 3 (HCM): JUP (p.Asp723_Tyr724del) ^1^	nr	VUS
			Case 4 (HCM): FLNC (p.Ala688Thr) ^2^	nr	VUS
			Case 5 (SUDS): MYBPC3 (p.Arg726Cys) ^3^	nr	LP
			Case 6 (SUDS): TTN (p.Thr34393Pro) ^4^	nr	VUS
			Case 7 (SUDS): MYBPC3 (p.Arg891 Alafs*160)	nr	P
			Case 8 (SUDS): MYPN (p.Trp7Glyfs*26) ^5^	nr	VUS
Marey et al. [49]	11 HCM (4)	+	Case 1: MYH7 (c.2105T>A)	nr	LP
Girolami et al. [50]	14 SCD (6)	-	Case 1: MYBPC3 (c.2441_2443del)	nr	LP
			Case 2: TNNT2 (c.517_519del)	nr	LP
			Case 3: MYH7 (c.2890G>C) ^6^	nr	VUS
			Case 4: ACTN2 (c. 1823G>A)	nr	VUS
			Case 5: TNNT2 (c.113C>T) ^7^	nr	VUS
			Case 6: TTN (c.57515_57517del) ^8^	nr	VUS
Kraoua et al. [51]	1 HCM/DCM	+	ACTN2 (c.355G>A)	nr	P
Fadoni et al. [52]	16 SCD (9)	+(2 HCM)	Case 1: MYBPC3 (c.2221delG); NEXN (c.1201delA)	FM/FM	P/VUS-NV
			Case 2: MYBPC3 (c.2221delG); DSG2 (c.370delT); NME1 (c.413delA)	FM/FM/FM	P/VUS-NV/VUS-NV
			Case 3: MYBPC3 (c.2221delG); MYH6 (c.5803delA)	FM/FM	P/VUS-NV
			Case 4: MYBPC3 (c.2221delG); ACTN2 (c.842delG); MYO6 (c.973delA)	FM/FM/FM	P/VUS-NV/VUS-NV
			Case 5: MYBPC3 (c.2221delG); MYH7 (c.2304delG);	FM/FM	P/VUS-NV
			Case 6: MYH7 (c.G2348T); NEXN (c.1201delA)	MM/FM	LP/VUS-NV
			Case 7: MYLK2 (c.C808T)	MM	VUS
			Case 8: ACAD9 (c.G976A)	MM	VUS
			Case 9: ACTN2 (c.C1330T); KCNE2 (c.T170C)	MM/MM	VUS/VUS
Tsaturyan et al. [53]	1 SCD	-	TPM1 (c.656A>T)	nr	DN-LP

nr: not reported; SM: splice mutation; MM: missense mutation; SiM: silent mutation; FM: frameshift mutation; NSM: nonsense mutation; B: benign; LP: likely pathogenic; P: pathogenic; NV: novel variant; VUS: variant of uncertain significance; DN: de novo variant. * Variant in a gene associated with altered ion channel function. ^ Linked to DCM. ^1^ Linked to ACM. ^2^ Linked to cardiomyopathy. ^3^ Linked to cardiomyopathy. ^4^ Linked to cardiomyopathy. ^5^ Linked to cardiomyopathy. ^6–8^ Linked to HCM and DCM.

## 4. Discussion

HCM is a complex disease that is commonly responsible for SCD, especially in young people, even though it is considered a morbidity potentially affecting any age. HCM shows marked heterogeneity in clinical features and prognosis, which creates a dilemma for physicians in setting up both diagnosis and treatment [54]. HCM is, also, a challenge for pathologists because of the great variability of phenotype, making the post-mortem diagnosis difficult, particularly in cases without anamnestic/clinical data. 

The macroscopic heart examination can offer findings that support the diagnosis of HCM. Asymmetrical or symmetrical hypertrophy is characteristic evidence [18,19,20,21,22,23,24,25,26,27]. The most observed hypertrophy is the asymmetric one, with a greater thickening of the ventricular septum compared to the left ventricular free walls, with a ratio of increase equal to 30% or more [55]. Regarding the asymmetric HCM, Teare [56] described the classical anatomic form, in which the septal thickening is at the basal anterior septum bulging beneath the aortic valve; thus, it determines the narrowing of the left ventricular outflow tract (LVOT) and anterior displacement of the papillary muscle (PM) and mitral leaflets [18,20,24,25]. This causes the systolic anterior motion of the mitral (SAM) valve during systolic contraction, as well as the mitral leaflet–septal contact, contributing to subaortic obstruction and, gradually, mitral regurgitation [57]. Moreover, the fibrosis at the septum subjacent to the aortic valve and subaortic mitral valve lesion can be observed [16,18,19,21,23]. However, some authors highlight that the hypertrophy of the basal anterior septum and the subaortic mitral valve lesion can be occasionally observed in other conditions, such as systemic hypertension, aortic valve stenosis, or “sigmoid septum” [58,59]; thus, the histological analysis is necessary to support the co-relation to HCM. Less common abnormalities of MV apparatus can be observed in the size, shape, and annulus angulation. Moreover, calcification may be found at the annulus and leaflets, as well as prolapse and clefts of the leaflets [60]. Chordae tendinae often appear fibrinous, with abnormal attachments to the leaflets and ventricular walls, and can undergo rupture [61]. Regarding papillary muscle, various abnormalities can occur, such as shortening, elongation, or thickening; abnormal attachments on the ventricular walls, or even directly to the mitral leaflets; additional heads or accessory PMs [60,62].

In some asymmetrical cases, the left ventricular hypertrophy can show morphologic variants, which are observed to be more prominent in other regions, such as the mid-ventricle, apex, anterior-lateral and posterior-basal free walls [63]. The prevalence of the symmetrical form varied from 10% to 42% [16,55], and it is characterized by the left ventricle concentric thickening with the reduction in the cavity size [19,20,22,23,24,25]. A recent study performed on a large sample (*n*. 2628) reported a prevalence of the main HCM morphological variances, as evaluated by cardiac MR, as follows: isolated basal septal hypertrophy in 46% of the subjects; reverse septal curvature in 38%; apical hypertrophy in 8% of the cases; mid-cavity obstruction with apical aneurysm in 3%; concentric HCM in 1%; and hypertrophy classified as other in 1% [64].

The apical aneurysm of the left ventricle (LVAA) represents a particular morphological variant that falls within the broad phenotypic spectrum of HCM. Its prevalence in subjects affected by hypertrophic cardiomyopathy is relatively low and is estimated to be between 2 and 5%; despite this, LVAA is probably underdiagnosed due to the intrinsic limitations of conventional echocardiography without contrast, which is commonly used for the follow-up [65]. The pathophysiological mechanisms that lead to the formation of these aneurysms are different, but the most relevant appear to be attributable to (i) systolic obstruction of the outflow tract of the left ventricle, with an increase in apical chamber pressure and a reduction in coronary perfusion pressure; (ii) the association between increased oxygen demand due to increased wall thickness and decreased oxygen supply due to reduced myocardial capillary density; and (iii) alterations of the microcirculation with a reduction in coronary reserve. These conditions, which can occur separately or simultaneously, are responsible for the onset of cardiac ischemic phenomena or true heart attacks, with the subsequent possible formation of silent aneurysms [66]. Apical aneurysms are increasingly recognized as an unfavorable prognostic indicator, as they appear to be associated with the onset of complications, including apical thrombus formation and thromboembolic stroke and arrhythmic events [67].

Together with asymmetric or symmetric left ventricle hypertrophy some cases reveal the involvement of the right ventricle with a variable degree of hypertrophy and cavity dimension decrease [18,19,23]; epidemiological data reported this association in 17.6% of the cases [68]. Typically, whitish or gray scars can be found in the hypertrophic myocardium and are often more pronounced in the septum [18,23], and they are related to fibrotic replacement. Interestingly, the fibrosis occurs in regions which are different from those of the major epicardial coronary competence territory; thus, their regional location could be a factor suggesting the presence of HCM.

The phenotypic spectrum of HCM can be further characterized by ventricular dilatation which is similar to dilated cardiomyopathy (DCM-like) [28,29,30]. It is considered to be the result of disease progression due to a marked fibrotic replacement of myocardial tissue, and the dilatation can affect the left ventricle or both ventricles with broad areas of fibrosis [69,70]. The dilatation can coexist with left ventricle hypertrophic sites [20,30]. The dilated phase of HCM is also known as the “burned-out” phase, and it occurs in approximately 10% of cases [70].

Microscopically, the HCM predominant features are the hypertrophy of myocyte, the myofiber disarray, and the fibrosis. The myocyte hypertrophy is common and a transverse diameter >40 µm (normal range 10–15 µm) has been reported [55]. The fibers also show the disarray pattern due to the architectural disorganization, in which the hypertrophic fibers are messily oriented perpendicularly or obliquely around a central core of collagen [18,21,25,68]. Moreover, hypertrophic myocytes have abnormal shapes with abnormal branch or triradiate and stellate forms [19,21]; the nuclei are bizarre and result in enlarged, hyperchromatic, or atypical shapes [19,21,23,24,25]. Even if disarray is not a pathognomonic sign of HCM, some studies describe related features that support the cardiomyopathy diagnosis. In particular, researchers reported a classification of the fiber disorganization into four classes, and they observed that the HCM cases had two predominant types [31]: small size areas of disarray in which adjacent cardiac muscle cells were aligned perpendicularly or obliquely to each other, usually forming tangled masses or “pinwheel” configurations (type I-A) and areas with relatively broad bundles of muscle cells (the cells were normally arranged) oriented at oblique or perpendicular angles to each other (type I-B). In this context, there are other important diagnostic considerations regarding the analysis of the amount of disarray as criteria for HCM assessment. Maron et al. [31] described the technique for the quantitative evaluation of myocyte disarray. Some studies suggested that the extension of disarray should be at least 5% in the relevant part of the tissue section (i.e., ventricular septum) [31,32], whereas other authors suggested that the disarray value should not be less than 10% [16,71]. Nevertheless, the literature reported that in HCM the amount of disarray is generally >20%, resulting in a sensitive and specific marker [31,68,72]; in one study, the mean percentage area of disarray in the ventricular septum was reported to be equal to 30 ± 16% [22].

The increased fibrosis is another finding of HCM that was described as having variable degrees from patchy to extensive and, frequently, as being more severe in the ventricular septum [18,19,20,21,22,23,24,25,26,27,73]. It is reported to be extensively severe in the left ventricle in the dilated phase of the cardiomyopathy [28,29,30].

HCM can be also characterized by the involvement of intramyocardial coronary arteries and small vessels, which present the proliferation of intimal and medial smooth muscle cells and collagen and are then responsible for wall thickening and lumen narrowing [18,22,23,29,30]. The authors reported that intramural artery changes were observed in more than 80% of HCM cases, especially at the interventricular septum [74]. These features seem to be more common in both cases with severe fibrosis and a dilated phase of HCM [22,75]. Regarding vascular abnormalities, myocardial bridging of the left anterior descending coronary may be observed [22]. The vascular changes can occur together with microscopic signs of subacute or acute myocardial ischemia [18,22,76]; in particular, the incidence of transmural myocardial infarction in the hearts of subjects who died from HCM was described as being up to 15% [77].

The abovementioned microscopic findings are considered to be the main substrate responsible for SCD, which is often the result of lethal arrhythmias. The arrhythmogenic role of myocyte disarray is well known due to the intercellular junction disorganization that negatively affects the electromechanical coupling [78]. The literature evidence highlights a greater incidence of premature SCD, which is probably related to malignant arrhythmia in the subject with extensive and severe disarray [79]. Also, fibrosis can contribute to the electrical instability which is responsible for SCD; in particular, it was reported that myocardial ischemia at any stage observed in HCM can determine a variable amount of fibrotic replacement [22]. The pathogenesis of myocardial ischemia was explained by several mechanisms mediated by intramural coronary arteries and small vessel disease or epicardial coronary myocardial bridging, in which the compromission of the blood flow can occur for both the obstruction determined by lumen narrowing and the transient compression, resulting in systolic contraction of the hypertrophied ventricle [69,80,81,82]. It is known that the increase in the myocardiocyte activity causes an increase in oxygen demand, especially in the hypertrophic regions, leading to cellular hypoxia. Despite that, although myocardial blow flow events are not always associated with the onset of ischemic events, their cyclical repetition throughout the life of these subjects can be considered the basis of the remodeling phenomena characterized by the post-ischemic fibrotic replacement [83,84]. Thus, the combination of myocardial disarray and ischemic damage is considered to be a malignant trigger for myocardial electrical instability and sudden death [22]. In some cases, death is due to heart failure, which is generally observed in the end stage of HCM (like dilated cardiomyopathy) for extensive myocardial fibrosis with marked dilatation of the ventricle [85].

Cardiac imaging using PMCT and PMMR, as also recognized by the Association for European Cardiovascular Pathology, can have an added value in contributing to the diagnosis of sudden cardiac death due to HCM [2,86]. A study analyzing cases of SCD underlined the usefulness of PMCT angiography, which can provide data suggesting the presence of HCM such as the left ventricular hypertrophy and the asymmetrical septal hypertrophy associated with hypervascularity in the upper portion of the septum [27]. Interesting evidence was also reported for PMMR, offering heart morpho-volumetric information (i.e., detailed data on cardiac wall thickening and weight), which can have a relevant applicative implication for the differential diagnosis between primary and secondary myocardial hypertrophy [34,35,36]. PMMR can provide other data suggesting HCM, such as the myocardial bridge, ventricular crypts, and anomalies of the papillary muscles and sub-mitral apparatus [35]. However, this investigation cannot be considered a substitute for autopsy findings being associated with a false positive; in fact, Puranik et al. [37] described, in one case, radiological evidence of mid-posterior wall hypertrophy, at the level of papillary muscle, that was not confirmed at autopsy. The effect of myocardial rigor mortis on wall thickness was also analyzed to improve the PMMR performance in HCM diagnosis. In the clinical setting, the HCM diagnosis is performed using the end-diastolic wall thickness of the left ventricle, which, in the post-mortem setting, is not feasible because of rigor mortis. In fact, the reserves of ATP in cardiac muscle, as well as in skeletal muscle, are consumed, and the heart may remain paralyzed (rigor mortis) in both early and mid-diastolic phases without any relation to the time interval between death and PMMR or to sex, age, cardiac weight, and body habitus [87]. Thus, a study suggested applying a standard deviation of wall thickness cut-off >2.4 as a diagnostic parameter in a forensic context [36].

The genetic test is a fundamental diagnostic investigation for the assessment of HCM. This cardiomyopathy has an inherited genetic trait, which is primarily autosomal dominant, in about half of cases [88]. Cohort studies reported that in about 40–60% of the cases, there is a single variant responsible for the disease [89]. There are also sporadic cases that can have a monogenic cause, which is possibly related to either an incomplete penetrance of a variant inherited from a parent or is due to de novo variants or, less commonly, to autosomal recessive inheritance [88].

In about the 10% of the cases, HCM is due to other genetic/non-genetic diseases, among which are malformation syndrome (i.e., Noonan and LEOPARD syndrome), metabolic disorders (i.e., Anderson–Fabry and Pompe disease), mitochondrial diseases, neuromuscular diseases, and infiltrative diseases (i.e., amyloidosis) [90]. In these cases, it is possible to detect other morphological alterations in the cardiac muscle and/or other organs and systems. Therefore, a multidisciplinary evaluation is necessary among pathologists, geneticists, and relevant specialists and remains crucial to reach an accurate post-mortem differentiation [91]. HCM is a relatively common finding in Noonan syndrome and is usually associated with both other cardiac anomalies, such as pulmonary valve dysplasia causing stenosis of the right ventricular outflow tract, atrial septal defect, and/or aortic bicuspid valve, and systemic anomalies, such as delayed growth, cryptorchidism, and/or blood clotting abnormalities. The genetic bases underlying the development of the pathology are also different: in HCM–Noonan-related forms, the most commonly detected mutation appears to be that of the RAF1 and PTPN11 genes [91,92]. Fabry disease is a recessive lysosomal storage disease, linked to the X chromosome, due to the deficiency of the enzyme α-galactosidase A (α-Gal A), which is secondary to the GLA gene mutation. This deficiency causes a progressive accumulation of globotriaosylceramide (GL-3) and related glycosphingolipids in cardiac, renal, neural, vascular, ocular and skin tissues. Cardiac involvement is characterized by a broad spectrum of signs and symptoms, including hypertrophic cardiomyopathy, which is associated with arrhythmias, coronary artery disease (primarily small vessel disease), and heart failure [93]. In cardiac amyloidosis (CA), fibrillar proteins are deposited at an extracellular level, interrupting the normal architecture and function of tissues, which then leads to the onset of phenotypic conditions such as left ventricular hypertrophy [94].

**Figure 2:**
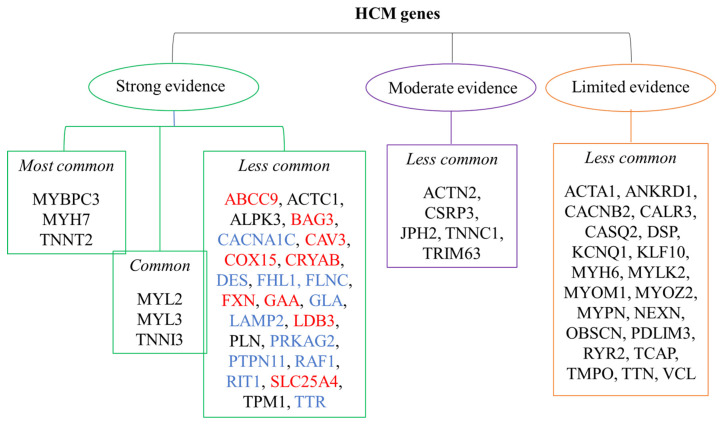
Frequency and strength of evidence of genes associated with HCM [11]; most common: >10% of the cases; common: 1–10% of the cases; less common: <1% of the cases; in red: genes associated with syndromes in which cardiomyopathy is not expected to occur as the only or presenting feature of the disease (ABCC9: Cantu syndrome; BAG3: Myofibrillar myopathy; CAV3: Caveolinopathy; COX15: Leigh syndrome; CRYAB: Alpha-B crystallinopathy; FXN: Friedreich ataxia; GAA: Pompe disease; LDB3: Myofibrillar myopathy; SLC25A4: Mitochondrial disease); in blue: genes associated with syndromes in which cardiomyopathy may be the only or presenting feature of the disease (CACNA1C: Timothy syndrome; DES: Desminopathy; FHL1: Emery–Dreifuss MD; FLNC: Myofibrillar myopathy; GLA: Anderson–Fabry disease; LAMP2: Danon disease; PRKAG2: PRKAG2 cardiomyopathy; PTPN11-RAF1-RIT1: Noonan syndrome; TTR: Transthyretin amyloidosis).

The Guidelines for the Management of Cardiomyopathies of the European Society of Cardiology (ESC) of 2023 report 57 genes associated with monogenic HCM [11,95]. These genes, with their frequencies and strength of evidence, are schematized in Figure 2. In particular, the genes with definitive/strong evidence of HCM co-relation are MYBPC3, MYH7 and TNNT2, which are reported as very commonly involved genes (>10% of tested cases); MYL2, MYL3, and TNNI3 are described as common (1–10% of tested cases) [96].

Thus, several cases are characterized by mutations in the genes encoding the structural components of the thick and thin filaments forming the cardiac muscle sarcomeres. The main genes encode for 11 sarcomeric proteins, showing >1400 mutations, affecting beta-myosin heavy chain, cardiac myosin binding protein C, cardiac troponin T, troponin I, alphatropomyosin, essential and regulatory myosin light chains, actin, alpha-myosin heavy chain, titin, and muscle LIM protein [97,98]; frequently, the genetic test in the clinical setting reveals that most patients have mutations at the first three genes while the other genes represent a small fraction. The mutations are generally missense with a single amino acid substitution; then, small deletions or insertions are described [16,99].

In this field, an important contribution belongs to the post-mortem genetic test, also known as the “molecular autopsy”, which provides data on the genes involved in HCM-related SCD and, particularly, on the several variants found at each gene that (in accordance with the American College of Medical Genetics and Genomics and the Association for Molecular Pathology Guidelines) [100] can be classified as pathogenic, likely pathogenic, of uncertain significance, likely benign, or benign. Thus, the scientific community describes the emerging value of the molecular analysis in such cases, which, furthermore, is the only tool to assess the cause of death in cases without well-defined structural heart abnormalities [2].

The results of the performed systematic review are in accordance with the previous evidence, which resulted in a major frequency of pathogenic or likely pathogenic mutation at the MYBPC3 [26,39,40,41,43,44,45,47,48,49,50,52] and MYH7 [38,39,40,41,43,44,46,47,48,49,50] genes; among the gene mutations responsible for the functional effect there were also TNNT2 [50], the other gene described as most commonly mutated in HCM and as having strong evidence of correlation; TPM1 [53], with strong evidence of HCM association, but with a frequency that was less common; and ACTN2 [51], a gene with moderate evidence of correlation and of being less commonly involved. Several different mutations were identified for each gene showing the allelic heterogeneity characterizing this disease [101]. Moreover, the review showed cases in which novel variants were found in the genes most commonly associated with HCM [44,52], supporting the contribution of the post-mortem genetic test to the extension of existing knowledge on the genetic basis of the disease.

Interestingly, in the included articles cases were described without clear heart structural alterations that revealed gene mutation with functional effects leading to the cardiomyopathy diagnosis [38,48,50,52,53]. Regarding the mechanism responsible for the HCM phenotype, several theories have been postulated, such as the “poison polypeptide” and the “null alleles” effects. These theories suggest the possibility of abnormalities on sarcomeric proteins, in which can occur the functional compromission or an impaired amount of the “wild type”, leading to both the alteration of the mechanical function of myocytes and cellular stress [16,101]. This may converge in the activation of the stress-response signal, determining the activation of myocyte transcriptional pathways and the compensatory tissue alterations (hypertrophy, disarray, and fibrosis) [102,103]. Moreover, to explain the SCD in cases without cardiac morpho-structural alterations, some authors suggested that the functional abnormalities of myocytes due to the sarcomere mutations may occur before the onset of gross myocardial hypertrophy, theoretically determining an increased risk of arrhythmia [50,104].

HCM research is also focused on the analysis of genotype–phenotype correlation with evidence belonging to both clinical and forensic/post-mortem studies; however, there are cases in which pathogenic or presumed pathogenic mutation were not identified in the subject with the HCM phenotype (for instance, in the clinical setting the identification of causal mutation has been reported in about 30% to 40% of patients) [98].

It was reported that about 5% to 7% of patients with HCM with ≥2 mutations seem to show a more severe phenotype (the so called “gene-dose theory”), as did the homozygous subjects [105,106]. The data obtained from the comparisons between the specific mutated genes showed variable results; for instance: the comparison between MYBPC3 and MYH7 did not reveal significant differences in cardiomyopathy phenotypical expression [107,108]; the comparison between the genes encoding the sarcomere thin filament (TNN2, TNNT3, TMP1 and ACTC) and the thick filament (MYBPC3, MYH7 and MYL2) concluded that there was an increased risk of advanced LV dysfunction and heart failure in cases with thin filament mutations, and an equal arrhythmic risk [109]. On the other hand, there are reports supporting the correlation of a specific variant in gene mutations and a more severe phenotype; p.Arg470Gln and p.Thr1046Met mutations in MYBPC3 have been reported to have an amount of myocyte disarray greater than 10%, even if two cases with the same variant (p.Thr1046Met) differed either for heart weight, percentage and distribution of disarray, or LV thickness [47]; p.Gly278GlufsTer22 and p.Phe295SerfsTer5 in MYBPC3 have been associated with higher heart weight-to-BMI ratios and death under the age of 40 [26]; Arg453Cys mutation in MYH7 has been described in an increased incidence of end-stage heart failure and premature death [110]. Despite several studies suggesting the association between specific gene mutations and reduced survival, this is not always true because of the great variation in phenotype expression observed in subjects having the same genetic variant. There is no evidence on genotype–phenotype correlation that is strong enough to provide information that is potentially useful for the patient care, especially regarding a phenotype prediction and the clinical outcomes. For this reason, the implementation of a model integrating a broad genetic profile and the deep clinical/morphostructural phenotyping may be the only tool for achieving effective findings and for implementing the patient prognostic perspective from the forensic point of view.

## 5. Conclusions

**Figure 3:**
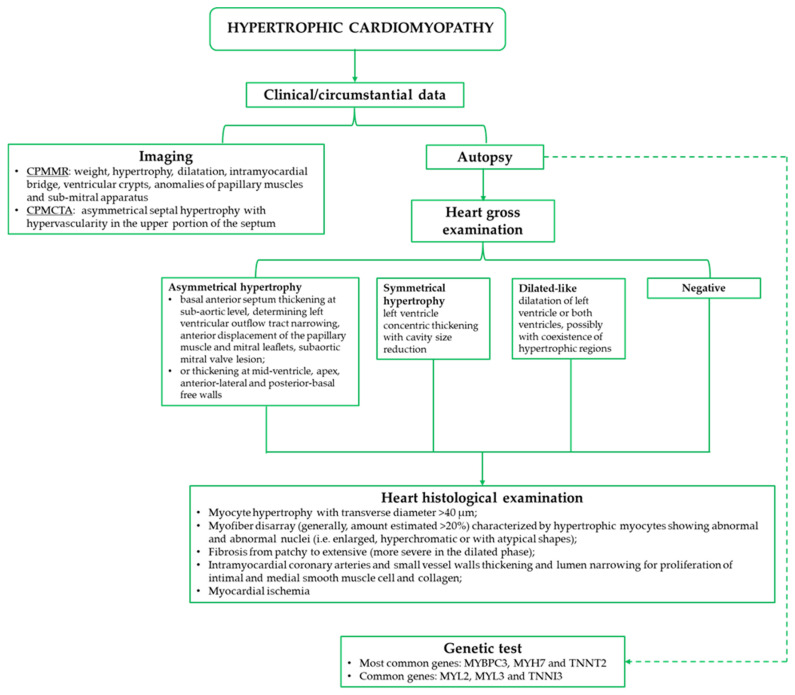
Synthesis of the main investigations and related findings for post-mortem diagnosis of HCM. CPMMR: cardiac post-mortem magnetic resonance; CPMCTA: cardiac post-mortem computed tomography angiography.

In conclusion, the forensic pathologist has a pivotal role in HCM diagnosis, which is not only limited to cases of SCD but also to those cases with positive clinical anamnesis, which refines the diagnosis. Whilst macroscopic findings may not be useful in reaching a certain diagnosis, the presence of “quantitatively effective” microscopic data on hypertrophy, myocytes disarray, fibrosis, and small vessel diseases can be considered confirmatory of HCM (Figure 3). Thus, the importance of performing a careful gross examination of the heart and an adequate histological sampling is of great importance, and, as suggested by the guidelines, the opinion of a cardio-pathology expert should be considered. These investigations are increasingly associated with the radiological ones, particularly the PMMR, which can provide additional information on cardiac morphology. Moreover, the literature reports the increasingly great importance of the post-mortem genetic test, especially (if considered from a forensic pathologist’s point of view) in those cases without clear cardiac morpho-structural alterations. In fact, in all those cases of SCD with negative macroscopic and histological findings, there would be the risk of not being able to reach a correct diagnosis. In these negative-phenotype forms, in which death could occur due to the onset of arrhythmias, the misdiagnosis of the pathology would not even allow the planned screening programs to be implemented. Nevertheless, the molecular autopsy should also be considered for the ethical and practical aspects related to the assessment of the genetic basis of the disease and, consequently, for the contribution to a better management of the relatives, both through genetic counselling and family screening.

## Data Availability

All the data are reported in the paper.
